# Optimal treatment of opioid induced constipation in daily clinical practice – an observational study

**DOI:** 10.1186/s12904-019-0416-7

**Published:** 2019-03-29

**Authors:** Elisabeth C. W. Neefjes, Hanneke van der Wijngaart, Maurice J. D. L. van der Vorst, Diederik ten Oever, Hans J. van der Vliet, Aart Beeker, Christiaan A. Rhodius, Hendrik P. van den Berg, Johannes Berkhof, Henk M. W. Verheul

**Affiliations:** 1 Department of Medical Oncology, Cancer Center Amsterdam, Amsterdam UMC, Vrije Universiteit, De Boelelaan 1117, 1081 HV Amsterdam, The Netherlands; 2 Department of Internal Medicine, Medical Center Alkmaar, Alkmaar, the Netherlands; 3 Department of Internal Medicine, Rijnstate Hospital, Arnhem, the Netherlands; 4 Department of Internal Medicine, Spaarne Gasthuis, Hoofddorp, the Netherlands; 5 Hospice Bardo, Hoofddorp, the Netherlands; 6 Department of Internal Medicine, Tergooi, Hilversum, the Netherlands; 7 Department of Epidemiology and Biostatistics, Amsterdam UMC, Vrije Universiteit, Amsterdam, the Netherlands

**Keywords:** Constipation, Opioid, Neoplasms, Methylnaltrexone, Laxatives, Pain management

## Abstract

**Background:**

Opioids are prescribed in over 40% of patients with advanced cancer, but side effects occur frequently. In this study we evaluated the development and treatment of opioid induced constipation (OIC), and OIC resolving effect of methylnaltrexone for different opioid subtypes in daily clinical practice.

**Methods:**

Patients with cancer using opioids were included in a retrospective chart analysis. Baseline characteristics, data on opioid use, laxative use, and OIC were collected. Patients with OIC who were prescribed methylnaltrexone, were included in a prospective observational trial (NCT01955213).

**Results:**

Thirty-nine of 327 patients (pts) with cancer who were treated with opioids suffered from OIC (overall prevalence 12%; 95%-CI: 8–15%). The prevalence of OIC was similar in patients treated with oxycodone or fentanyl (12 of 81 pts. vs. 18 of 110 pts., RR 0.9; 95%CI 0.4–2.0). The morphine equivalent daily dose did not significantly differ between opioid subtypes (fentanyl 89 mg (IQR 60–180) vs. oxycodone 40 mg (40–80), *P* = 0.231). Twenty-two individual patients (7%) were admitted for OIC. Most effective laxatives in admitted patients were enemas, methylnaltrexone, or 4-l polyethylene-glycol solution. In the prospective observational study, the effect of methylnaltrexone could be evaluated in 23 patients. Eleven patients achieved the primary endpoint of ≥2 laxation responses out of the first four doses methylnaltrexone, independent of opioid subtype.

**Conclusions:**

OIC is a burdensome clinical problem independent of opioid subtype. Timely intensification of prophylactic laxative treatment, especially when opioid doses increase, may help to prevent OIC. Clinically overt OIC requires a more intensive laxative regimen with for example methylnaltrexone.

**Trial registration:**

NCT01955213.

## Background

Over 40% of patients with advanced cancer need opioids at some point during their disease trajectory [1, 2]. Opioids may cause transient side effects such as nausea and drowsiness [3]. A non-transient side effect of opioids is constipation [3]. Opioid induced constipation (OIC) is caused by binding of opioids on the μ-receptor in the intestines, leading to a decrease in peristaltic movements and a longer transit time, allowing more water absorption from the stool [4]. Differences in bio distribution and opioid receptor binding profile result in varying incidence rates of OIC between different opioid subtypes [5]. While patients included in trials are frequently prescribed single opioids, in daily practice, combinations of different opioid subtypes are used to tailor pain management and to optimize ease of use. Different laxatives are also prescribed in variable doses in line with international guidelines advising to prescribe prophylactic laxatives when opioids are being used [6, 7]. Most used laxatives are osmotic agents e.g. polyethylene glycol (PEG) or magnesium oxide, and stimulant laxatives e.g. bisacodyl. Adherence to the guidelines by physicians varies, [8] and patients frequently stop using these prophylactic laxatives due to diarrhea [9, 10]. Even more, other patient- or treatment-related factors may cause constipation in patients with cancer using opioids, such as reduced fluid intake, or the use of 5-HT3 receptor antagonists for chemotherapy induced nausea and vomiting. Real life data on the prevalence of constipation, and more specific opioid induced constipation, in patients with cancer using opioids are scarce, [10–12] and comparative trials evaluating different treatment options for OIC have not yet been conducted.

The optimal treatment strategy for constipation depends on its cause in an individual patient [6]. Various strategies to treat OIC are available, including to start, add, or increase the dose of laxatives; to admit patients to the hospital for more intensive laxation treatment; to decrease the dose of opioids (which is often not feasible); to switch to another opioid subtype; or to use various formulations of the μ-opioid receptor antagonist naloxone [7, 13]. Naloxone has been used in the past for opioid induced constipation, but its use was associated with opioid withdrawal and decreased pain relief [14]. As an alternative methylnaltrexone, a quaternary amine of naltrexone that does not cross the blood-brain barrier, can be prescribed. It can be used as a peripheral μ-receptor antagonist which resolves OIC in 50% of patients, without influencing the analgesic effect achieved by the central effects of the opioid [14, 15].

In this study we evaluated the development and treatment of opioid induced constipation in daily clinical practice, and the OIC resolving effect of methylnaltrexone for different opioid subtypes. We performed a retrospective chart review evaluating real-life data on opioid use and the incidence of OIC in every day clinical practice, and the effect of different treatment options for OIC. In addition, a prospective observational trial was set up to study the effect of methylnaltrexone for OIC caused by different opioid subtypes, in order to determine which patients could benefit most from this treatment [5].

## Methods

### Retrospective chart review

Patients who were prescribed opioids by their oncologist at an academic hospital and a large teaching hospital over a six month period in 2014 were included in the retrospective study. Data concerning baseline characteristics, opioid and laxative use, and the occurrence of OIC were extracted from the patient charts. When multiple opioid prescriptions were made in the study period, the prescription with the highest oral morphine equivalent daily dose (MEDD) was used for this analysis. Conversion rates from buprenorphine, fentanyl, subcutaneous morphine, oxycodone and tramadol to oral morphine were 2.29, 2.4, 3, 3, and 0.2, respectively [16]. For methadone conversion rates to oral morphine were 4 for doses < 22.5 mg, 6 for doses between 22.5 and 50 mg, and 8 for doses > 50 mg [16]. Notes on the patient’s laxation pattern in the period between 30 days before and after the selected opioid prescription were extracted to evaluate if OIC had occurred. Admittances for OIC were registered for the year prior to and following the selected opioid prescription. In order not to miss admittances with OIC as an underlying cause, we reviewed each admission for the mentioning of (opioid-induced) constipation as a underlying cause for the reason of admission. Constipation was defined as a reported change in laxation frequency to less than 3 stools per week. Due to the retrospective nature of this part of the study, subjective components to constipation could not be assessed. Constipation was considered as opioid induced if this was made explicit in the patient chart, and/or if there was no other major contributing factor specified (e.g. dehydration, obstruction of the GI-tract by a tumor or carcinomatous peritonitis, or side effects from other drugs).

### Sample size and statistics

Most of the presented data are descriptive. It was expected that, even despite prophylactic laxative use, patients using fentanyl would have a lower incidence rate of OIC than patients using oxycodone [5]; 10% of the patients using fentanyl would develop OIC, compared to 30% of the patients using oxycodone. In a population in which 40% used fentanyl and 25% oxycodone, a study sample with at least 120 patients using fentanyl and 80 using oxycodone was needed to have 90% power to detect the hypothesized difference in the incidence of OIC between fentanyl and oxycodone (*P* < 0.05 was considered statistically significant). Descriptive data are presented by their mean or median, and SD or inter quartile range (IQR). Comparisons were made with a Pearson’s Χ^2^ test or Mann-Whitney U test, as considered appropriate. For assessing the effect of multiple factors, multiple logistic regression was used.

### Prospective observational study

The methods of the observational trial to determine the differences in the effect of methylnaltrexone on OIC induced by different opioid subtypes are published elsewhere [5]. Briefly summarized, patients with OIC caused by morphine, oxycodone, or fentanyl, without contra-indications for methylnaltrexone, received a 14-day treatment with methylnaltrexone subcutaneously (SQ) every other day. In this period a laxation diary was kept to monitor the effect of methylnaltrexone. The Bowel Function Index questionnaire (BFI) [17] was completed at the start and end of this treatment period. We aimed to include 78 patients in the oxycodone group, 78 patients in the morphine group and 39 patients in the fentanyl group. This study was approved by the Medical research Ethics Committee of the VU University medical center and performed in accordance with the Helsinki Declaration of the World Medical Association.

### BFI questionaire

The BFI consists of three questions about symptoms of constipation experienced during the past week. Answers to these questions are rated on a scale from 0 to 100 and the final score is calculated by the mean of the three answers. A decrease of 12 points or more between start and end of the study is thought to be a clinically significant response [5, 17].

### Software for data storage and analysis

Data of both studies were anonymized and stored in the web based database system Open Clinica (Open Clinica version 3.3). Statistical analyses were performed with IBM SPSS version 22.0 (IBM, Armonk, NY, USA).

## Results

### Patient characteristics

Three hundred twenty seven patients were included in the retrospective analysis. Characteristics of these patients are presented in Table 1. Fentanyl (*N* = 131, 40%) and oxycodone (*N* = 90, 28%) were the most frequently prescribed opioid subtypes. Ninety-four patients (29%) did not use a maintenance opioid, only rescue doses. Tumor types, treatment intention (curative or palliative), intake failure (especially reduced fluid intake), and abdominal innervation problems (paralysis of the intestines) between the fentanyl and oxycodone group were significantly different. For fentanyl maintenance treatment continuous-release patches were used. These were mostly prescribed for patients treated with chemo-radiotherapy for head and neck cancer, which explains most differences in characteristics between the treatment groups. When correcting for this tumor type, only intake failure remained statistically significant more frequent in the patients using fentanyl patches (28/95 vs. 8/85 in the oxycodone group, *P* = 0.001), and abdominal innervation problems were more frequent in the oxycodone group (7/85 vs. 1/95 in the fentanyl group, *P* = 0.017).

**Table 1:** Patient characteristics - retrospective chart review

		Total *N* = 327 (%)	Fentanyl Maintenance *N* = 131 (%)	Oxycodon maintenance *N* = 90 (%)	*P* (fentanyl vs. oxycodone)
Age	mean (SD)	63 (12)	64 (11)	63 (12)	0,368
Sex	Male	146 (45)	64 (49)	41 (46)	
	Female	181 (55)	67 (51)	49 (54)	0,629
Cancer type	Gastro-intestinal	110 (34)	43 (33)	26 (29)	
	Breast	54 (17)	13 (10)	19 (21)	
	Genito-urethral	62 (19)	23 (20)	26 (29)	
	Skin	15 (5)	7 (5)	5 (6)	
	Lung	1 (0)	1 (1)	0 (0)	
	Head&Neck	53 (16)	33 (25)	5 (6)	
	Brain	3 (1)	1 (1)	0 (0)	
	Sarcoma	6 (2)	2 (2)	2 (2)	
	Other	23 (7)	5 (4)	7 (8)	0,005*
Treatment intention	Curative	71 (22)	37 (28)	9 (10)	
	Palliative	256 (78)	94 (72)	81 (90)	0,001*
Current Treatment	Follow-up	7 (2)	3 (2)	1 (1)	
	Watchfull waiting	22 (7)	7 (5)	5 (6)	
	Chemotherapy	90 (28)	32 (24)	28 (31)	
	Radiotherapy	15 (5)	7 (5)	5 (6)	
	Chemoradiotherapy	38 (12)	25 (19)	3 (3)	
	Targetted therapy	51 (16)	17 (13)	17 (19)	
	Best supportive care	81 (25)	33 (25)	24 (27)	
	Other	23 (7)	7 (5)	7 (8)	0,032*
ECOG performance status	0	14 (4)	3 (2)	7 (8)	
	1	110 (34)	40 (31)	36 (40)	
	2	67 (21)	22 (17)	20 (22)	
	3	29 (9)	14 (11)	11 (12)	
	4	17 (5)	6 (5)	1 (1)	
	Unknown	90 (28)	46 (35)	15 (17)	0,264
Contributing factors	Peritoneal tumor depositions	52 (16)	21 (16)	13 (14)	0,748
	Intestinal metastasis	19 (6)	8 (6)	3 (3)	0,352
	Involvement of other abdominal organs	115 (35)	45 (34)	34 (38)	0,602
	Hypercalcemia	21 (6)	13 (10)	6 (7)	0,239
	Reduced mobility	116 (36)	47 (36)	34 (38)	0,773
	Intake failure	71 (22)	41 (31)	9 (10)	< 0,001*
	Constipating co-medication	75 (23)	30 (23)	18 (20)	0,607
	Abdominal innervation problems	11 (3)	1 (1)	7 (8)	0,006*
	Bowel disease	4 (1)	1 (1)	1 (1)	0,789
	Depression	3 (1)	2 (2)	0 (0)	0,239
Pain Score^a^	NRS 0–10, mean (SD)	4,0 (6,0)	4,0 (5,0)	5,0 (6,5)	0,948
Opioid doses^b^	Maintenance dose, median (IQR)	60 (29–120)	60 (29–120)	40 (40–80)	0,006*
	Rescue dose^c^	20 (3–40)	23 (10–50)	20 (0–40)	0,259
Rescue opioid subtypes	No rescue opioid	36 (11)	9 (7)	6 (7)	
	Buprenorfine	14 (4)	0 (0)	0 (0)	
	Fentanyl	24 (7)	17 (13)	3 (3)	
	Methadon	2 (1)	0 (0)	0 (0)	
	Morphine	57 (17)	34 (26)	5 (6)	
	Oxycodon	185 (57)	71 (54)	76 (84)	
	Tramadol	5 (2)	0 (0)	0 (0)	
	Other	4 (1)	0 (0)	0 (0)	< 0,001*

* marks the statistically significant results

^a^Pain score at a numerical rating scale (NRS) from 0 to 10 at the moment the opioid was prescribed (*N* = 115)

^b^Oral morphine equivalent daily doses (MEDD) at the moment of opioid prescription

^c^Rescue opioid doses, calculated for patients with a known rescue frequency (total *N* = 135, fentanyl *N* = 64, oxycodone *N* = 30)

Sixty percent of the patients used concurrent laxatives, mostly a polyethylene-glycol solution (PEG), magnesium oxide, and/or enema’s (55, 7, and 6% respectively, Table 2). Thirty-one patients (9%) used two or more types of laxatives. There were no statistically significant differences in the number of patients using a specific laxative, nor in the dose of laxatives, between patients using fentanyl or oxycodone, except for the dose of magnesium which was higher in patients using oxycodone (*N* = 5) than in patients using fentanyl (*N* = 13) (median 6 vs. 3 tablets per day, *P* = 0.033).

**Table 2:** Laxative use at opioid prescription

	Total *N* = 327 (%)	Fentanyl maintenance *N* = 131 (%)	Oxycodone maintenance *N* = 90 (%)	*P* (fentanyl vs. oxycodone group)
Any laxative	195 (60)	88 (67)	63 (70)	0,657
Polyethylene glycol solution	181 (55)	78 (60)	60 (67)	0,283
PEG dose, median^a^	1	1	1	
Magnesium	23 (7)	13 (10)	5 (6)	0,243
Magnesium dose, median^b^	3	3	6	
Bisacodyl	3 (1)	3 (2)	0 (0)	0,148
Bisacodyl dose, median^c^	1	1	.	
Lactulose	7 (2)	3 (2)	2 (2)	0,973
Lactulose dose, median^d^	1	1	2	
Enema’s	18 (6)	5 (4)	7 (8)	0,202
Enema frequency, median^e^	1	1	1	
Other	3 (1)	2	0 (0)	0,239

^a^Number of polyethylene glycol sachets per day. Sachet à 13,7 g containing polyethylene glycol and elektrolytes (potassiumchloride, sodiumchloride, sodium hydrogen carbonate). Inter quartile range (IQR) total group: 1–2; fentanyl group: 1–2; oxycodone group: 1–2

^b^Number of magnesium oxide tablets à 500 mg per day. IQR total group: 2–6; fentanyl group 2–3; oxycodone group: 4–6

^c^Number of bisacodyl tablets à 5 mg per day. Range total group: 1–1; fentanyl group: 1–1; oxycodone group: not applicable

^d^Number of lactulose doses per day. One dose equals 15 ml of lactulose 670 mg/ml. Range total group: 0–2; fentanyl group: 0–2; oxycodone group: 0–2

^e^Number of enema’s per day, usually a sodium phosphate enema of 133 ml containing 31,8 mg sodium acid phosphate and 139,1 mg sodium dihydrogenphosphate per ml. IQR total group: 0,5–1; fentanyl group: 0–1; oxycodonde group: 0,5–1

When converted to oral morphine equivalent daily dose (MEDD), the median dose of regularly taken opioids was 60 mg (IQR 29–120). Two-hundred-and-ninety-one patients (89%) were prescribed rescue opioids, mostly short-acting oxycodone (*n* = 185, 57%).

### Opioid induced constipation

For 257 patients (79%) the attending physician made a note in the patient’s chart on their laxation pattern. Sixty-eight patients (21%) were constipated, of whom 39 (12%) were considered to be constipated due to opioids (Table 3). OIC was equally prevalent in patients treated with oxycodone or fentanyl (12 of 81 pts. vs. 18 of 110 pts., RR 0.9, 95% CI 0.4–2.0). Although there was a statistically significant difference in the oral morphine equivalent daily dose (MEDD) of regularly taken opioids between patients using fentanyl and oxycodone at opioid prescription (fentanyl MEDD 60 mg (IQR 29–120), oxycodone MEDD 40 mg (IQR 40–80), *P* = 0.006), the difference in the MEDD at diagnosis of OIC was not statistically significant (fentanyl 89 mg (IQR 60–180) vs. oxycodone 40 mg (IQR 40–80), *P* = 0.231). When analyzing the effects of opioid subtypes fentanyl and oxycodone and MEDD on OIC in a multiple logistic analysis, none showed statistical significance (OR for the risk at OIC for MEDD 0.997 (95% CI 0.990–1.005); for fentanyl 1.310 (95% CI 0.351–4.892); and for oxycodone 4.086 (95% CI 0.630–26.495)). Also, no difference in OIC was found between patients using oxycodone or fentanyl as rescue opioid (13% vs. 9%, *P* = 0.554).

**Table 3:** Opioid Induced Constipation

Opioid maintenance type^a^	*N*=	Note on laxation pattern *N* (%)^b,c^	Morphine dose (MEDD)^d^ median (IQR)	Constipation^e^*N* (%)	MEDD^d,f^ median (IQR)	Opioid Induced Constipation^g,h^ *N* (%)	MEDD^i^ median (IQR)	Admission for OIC^j^ *N* (%)^k^	MEDD^l^ median (IQR)
No maintenance opioid	94	77 (82)	0 (0)	12 (13)	0 (0)	1 (1)	0 (0)	1 (1)	0
Buprenorfine	16	16 (100)	105 (31–180)	5 (31)	24 (17–98)	3 (19)	30 (17–120)^m^	2 (13)	54 (17–90)^o^
Fentanyl	110	86 (78)	89 (60–150)	25 (23)	89 (60–180)	18 (16)	89 (60–180)	11 (10)	180 (60–180)
Methadon	3	2 (67)	60 (60–240)^m^	2 (67)	120 (60–240)^m^	1 (33)	240 (0)	0 (0)	n.a.
Morphine	5	3 (60)	60 (40–96)	3 (60)	60 (40–96)^m^	2 (40)	68 (40–96)^m^	3 (60)	60 (40–80)^o^
Oxycodon	81	64 (79)	40 (40–80)	19 (23)	40 (40–80)	12 (15)	40 (40–80)	5 (6)	60 (40–140)
Tramadol	14	7 (50)	30 (20–40)	1 (7)	20 (0)	1 (7)	20 (0)	0 (0)	n.a.
Other	4	2 (50)	7 (5–10)^m^	1 (25)	n.a.^n^	1 (25)	n.a.^n^	0 (0)	n.a.
Total	327	257 (79)	60 (40–120)	68 (21)	60 (40–120)	39 (12)	60 (40–120)	22 (7)	70 (40–180)

^a^Opioid maintenance type at note on laxation pattern or at prescription (if no note was found in the patient records)

^b^Number of patients in each subtype for whom a note was made at their laxation pattern, *P* = 0.931

^c^Percentage of patients with a note on their laxation pattern for each opioid subtype

^d^Oral Morphine Equivalent Daily Dose (MEDD) in milligrams

^e^Number of patients with a diagnosis of constipation in each opioid subtype, *P* = 0.030

^f^The difference between the fentanyl group and the oxycodone subgroup was statistically significant, *P* = 0.043

^g^Number of patients with a diagnosis of opioid induced constipation (OIC), *P* = 0.006

^h^Percentage of patients with a diagnosis of opioid induced constipation for each opioid subtype

^i^The difference in MEDD of patients using fentanyl or oxycodone was not statistically significant, *P* = 0.231

^j^Opioid subtype the papient was using at admission for OIC

^k^Percentage of patients admitted for OIC relative to the number of patients using this opioid subtype at the moment of the opioid prescription included in this analysis

^l^Oral Morphine Equivalent Daily Dose (MEDD) at the moment of admission for OIC; IQR = inter quartile range

^m^Range instead of interquartile range, due to low number of patients using this opioid subtype

^n^The MEDD could not be calculated because the conversion rate for this opioid subtype is unknown

^o^Range in stead of inter quartile range, due to the low number of patients using this opioid subtype

In the majority of the patients with OIC, the opioid dose remained stable (*N* = 14) or was increased (*N* = 14). In 32 patients diagnosed with OIC (82%) the laxative treatment was adjusted, mostly by prescribing a new laxative (*N* = 20). The main reason not to alter the laxative treatment while a patient was diagnosed with OIC, or to even stop the laxative treatment, was that the patient had entered the terminal phase and was not able to take oral medication anymore (*N* = 6).

### Admission for OIC

Twenty-two individual patients (7%) were admitted to the hospital for OIC (Table 3). The median MEDD at admission was 70 mg (IQR 40–180). Eleven patients used fentanyl, with a median MEDD of 180 mg (IQR 60–180). Five patients used oxycodone, with a median MEDD of 60 mg (IQR 40–140). The difference between the doses of fentanyl and oxycodone at admission was not statistically significant (*P* = 0.145). The opioid prescription was adjusted because of OIC in 4 of the 22 patients; in one patient the dose was lowered, in three patients an opioid rotation was performed.

Patients admitted for OIC were mostly treated with enema’s (*N* = 16) and a 4-l polyethylene glycol solution (*N* = 11). Complete resolution of constipation was seen with enema’s (*N* = 5/16), methylnaltrexone SQ (*N* = 2/5) and with a 4-l polyethylene glycol solution (*N* = 2/2).

### Effect of SQ methylnaltrexone in different opioid subtypes

Twenty-six patients were included in the methylnaltrexone trial between July 2012 and December 2015. Baseline characteristics of the included patients are presented in Table 4. Sixteen patients used oxycodone, eight fentanyl and two morphine. Three patients were lost to follow-up because the laxation diary was not returned (two in the fentanyl group, one in the oxycodone group). Of the 23 patients who returned response data of at least one dose of SQ methylnaltrexone, 11 met the primary endpoint of at least 2 laxation responses out of the first 4 doses. These were 8 patients in the oxycodone group (8 of 15 pts., 53%), 2 in the fentanyl group (2 of 6 pts., 33%) and 1 in the morphine group (1 of 2 pts., 50%). Reasons to prematurely stop SQ methylnaltrexone treatment were: per patient request (*N* = 4), adverse events (*N* = 2: 1 diarrhea, 1 abdominal pain), intercurrent illness (*N* = 1), death due to disease progression (*N* = 1), and unknown (*N* = 1).

**Table 4:** Patient characteristics methylnaltrexone trial

		Total *N* = 26	Oxycodone group *N* = 16	Fentanyl group *N* = 8	Morphine group *N* = 2
Age	mean (SD)	59 (13)	58 (10)	58 (18)	62 (18)
Sex	Male	11	9	2	0
	Female	15	7	6	2
Treatment setting	Hospital	20	11	7	2
	Ambulant	6	5	1	0
Cancer Type	Gastro-intestinal	9	6	3	0
	Breast	6	3	2	1
	Genito-urethral	6	5	1	0
	Other	5	2	2	1
Concomitant laxatives	PEG	19	12	5	2
	Magnesiumoxide	7	4	3	0
	Other	3	3	0	0
Patients using > 1 laxative		4	3	1	0
Morphine equivalent daily dose	median (range)	80 (40–540)	80 (40–160)	120 (60–240)	525 (510–540)

*PEG* polyethylene glycol solution

### Bowel function index (BFI)

Fourteen patients completed the BFI questionnaire on day 0 and 14 (2 in the fentanyl group, 1 in the morphine group, and 11 in the oxycodone group). The median change between start and end of study was − 5.8 points (IQR − 42.1 to 13.8), where a 12-point decline was previously defined as a clinically significant improvement. There were no statistically significant differences in BFI changes between patients who did or did not meet the studies primary endpoint (median ΔBFI 0.8 vs. -6.7 respectively, *P =* 0.573).

## Discussion

To our knowledge, this is the first study in which the frequency of OIC in real life in an unselected population of patients with cancer is being evaluated. In a study by Wirz et al. [10] and a survey by Bell et al. [12] among long term opioid users with a wide range of diseases data on this subject were limited to secondary endpoints in clinical trials. Bell et al. found that 81% of the 322 included patients (a selection from 130,293 originally invited patients) suffered from constipation despite laxative use, but it remained unclear to what extent the constipation was opioid induced. Wirz et al. performed a prospective observational study in ambulatory patients with cancer referred to the pain clinic. This study revealed a 5,7% incidence rate of constipation in a highly selected group of ambulatory patients using stable opioid doses > 28 days, without the need for break-through opioids, and who were not undergoing chemo- or radiotherapy treatment [10].

In our retrospective chart review 21% of the 327 patients with cancer using opioids suffered from constipation, which was opioid induced in 12% (39 patients). Eighty-seven percent of these patients (34 of 39 pts) developed OIC despite laxative use. The doses of prophylactic laxatives in this study group were relatively low (e.g. a single polyethylene glycol solution once per day). Therefore, intensification of the prophylactic laxative treatment, especially when the opioid dose increases, might help to reduce the number of admissions for OIC. However, when a patient is admitted for OIC, dose increases of these laxatives do not seem to resolve the problem. At this point more intensive treatments, such as a 4-l polyethylene glycol solution, enema’s, and possibly SQ methylnaltrexone, are more effective.

Our hypothesis that OIC occurs more often in patients using oxycodone than in patients using fentanyl, was not confirmed. Although the median MEDD of patients using fentanyl was more than twice the median MEDD for the patients using oxycodone at the moment patients were diagnosed with OIC, this difference was not statistically significant. Therefore, we cannot conclude that oxycodone is more constipating than fentanyl. It might be that the large proportion of patients using oxycodone as rescue opioid next to fentanyl as maintenance opioid has influenced the results, but there were no statistically significant differences found between patients using different subtypes of rescue opioids. This mixture of opioid subtypes reflects real daily practice, which is one of the strengths of this study. Our data complement the findings from opioid and laxative registration studies by providing valuable information on treatment practices and patient characteristics among unselected patients. This information is necessary to guide treatment decisions and for reimbursement and payment decisions.

The results of the chart review are limited to the notes that were made in the patient charts and the hospitals electronic prescription systems. Therefore it is possible that some cases of OIC were missed. Also, data on the actual opioid intake were not available. Based on the low number of records for pain scores noted at the moment of opioid prescription (*N* = 115 of 327 pts), and the low number of records of the frequency of rescue opioid use (*N* = 135 of 327 pts), there might be a gap between what is discussed by the patient and the physician, and what is noted in the patient’s chart. Other measures to prevent OIC, such as life-style advises, that were discussed with the patient, were not assessed in this study because they were often not recorded in the patient’s chart, and compliance to these advises could not be assessed. Another limitation is that most physicians only diagnosed constipation based on a reduced stool frequency. More subjective components of constipation, such as a feeling of abdominal distension, or straining to pass the stool, were given less attention.

The prospective observational study evaluating the efficacy of SQ methylnaltrexone in different opioid subtypes did not reach the anticipated sample size. The results of this trial therefore do not have enough power to draw any conclusions with regard to the hypothesis that patients using fentanyl would have a lower response rate to SQ methylnaltrexone than patients using oxycodone or morphine. The low inclusion rate was due to the significantly lower than expected prevalence of OIC, and the fact that many patients used a combination of different opioid subtypes, which was an exclusion criterion for the study. Contra-indications for SQ methylnaltrexone use were rare. Only 10 patients received all seven methylnaltrexone administrations. The others stopped treatment prematurely due to side effects or the lack of efficacy. There were also a number of patients who requested to stop treatment even though it was effective. These patients did not suffer from grade 3 side effects, but generally reported a feeling of unease during the first hours after methylnaltrexone administration that may be interpreted as mild side effects or mild opioid withdrawal symptoms. These observed side effects are important for designing possible future studies looking into the effect of μ-opioid receptor blockade on tumor progression [18].

For clinical practice the results of this study indicate that it is important to ask patients who use opioids regularly about their laxation pattern, and to adjust the prophylactic laxative prescription with increasing opioid doses. Thereby the development of OIC could possibly be prevented or managed in an ambulant setting, instead of requiring a hospital admission.

## Conclusions

In this real-life cohort of patients with cancer, OIC was encountered in 12 % of the patients using opioids and lead to a hospital admission in 7 %. Timely intensification of prophylactic laxative treatment, especially when the opioid dose increases, might help to further reduce the number of patients with cancer suffering from OIC and may help to prevent hospital admission. Treatment of OIC, once it has developed, requires a more intensive laxative regimen with for example SQ methylnaltrexone, independent of opioid subtype.

## Abbreviations

BFI: Bowel Function Index
IQR: Inter quartile range
MEDD: Oral morphine equivalent daily dose
OIC: Opioid induced constipation
OR: Odds ratio
PEG: Polyethylene glycol solution
